# Surgical interventions for simple phakic fovea-splitting rhegmatogenous retinal detachment: a comparative study of scleral buckling and pars plana vitrectomy

**DOI:** 10.3389/fmed.2024.1537416

**Published:** 2025-01-10

**Authors:** Haiqin Zhu, Qintuo Pan, Zhaoliang Zhang, Zongduan Zhang, Xiaoyin Ma, Xuting Hu

**Affiliations:** ^1^National Clinical Research Center for Ocular Diseases, Eye Hospital, Wenzhou Medical University, Wenzhou, China; ^2^State Key Laboratory of Ophthalmology, Optometry and Visual Science, Eye Hospital, Wenzhou Medical University, Wenzhou, China

**Keywords:** fovea-splitting, rhegmatogenous retinal detachment, surgical interventions, scleral buckling, pars plana vitrectomy

## Abstract

**Aims:**

To compare the efficiency of scleral buckling (SB) and pars plana vitrectomy (PPV) with or without SB in patients with primary simple phakic fovea-splitting rhegmatogenous retinal detachment (RRD).

**Methods:**

A retrospective case–control study included 101 patients aged <55 years diagnosed with phakic fovea-splitting RRD. The primary outcome was functional success, defined as achieving a postoperative logarithm of the minimum angle of resolution best-corrected visual acuity of 0.3 or better at 6 months post-surgery. Secondary outcomes included primary and final anatomical success and postoperative complications.

**Results:**

Fifty-one eyes underwent SB, and 50 eyes underwent PPV. In the SB group, 31 eyes (60.8%) achieved functional success compared with 22 eyes (44.0%) in the PPV group (*p* = 0.091). There was no significant difference in the primary anatomical success (SB = 94.1%, PPV = 94.0%) and final anatomical success (SB = 100%, PPV = 100%). The incidences of ocular hypertension, epiretinal proliferation, cystoid macular edema, and persistent subretinal fluid in the SB group were 37.3% (*p* = 0.059), 5.9% (*p* = 0.034), 3.9% (*p* = 0.051), and 74.5% (*p* < 0.001), respectively, whereas in the PPV group they were 56.0, 20.0, 16.0, and 22.0%, respectively. In multivariable analyses, PPV was significantly associated with epiretinal proliferation formation (OR: 4.000, 95% CI: 1.030–15.534, *p* = 0.045).

**Conclusion:**

SB may offer comparable outcomes to PPV in managing phakic fovea-splitting RRD, and careful surgical technique selection is advised due to postoperative complications.

## Introduction

Rhegmatogenous retinal detachment (RRD) is a critical cause of visual impairment, with an annual incidence rate of approximately 12 individuals per 100,000, which is increasing (1). Predictors of poor visual outcomes prior to surgery include advanced age (2, 3), fovea-off RRD (4), proliferative vitreoretinopathy (PVR) (2, 3), vision loss for over a week (2, 3), poor preoperative visual acuity (VA) (2, 5), extensive detachment (5), and vitrectomy-based procedures (5). Despite the high success rates of anatomical reattachment achieved through modern vitreoretinal surgical techniques, visual outcomes for fovea-off RRD have not improved proportionally (6).

Fovea-splitting RRD, a subtype identified on optical coherence tomography (OCT) images, involves detachment of the retina up to the fovea without complete detachment (7, 8). The incidence of fovea-splitting RRD is approximately 2.4% in patients with RRD (9). Patients with fovea-splitting RRD exhibit preoperative VA that is intermediate between those with fovea-on and fovea-off RRD (7, 8). However, their final visual acuity is similar to those with fovea-on RRD (4, 8), and is not influenced by the timing of surgery (4, 9, 10). The unique anatomical involvement of the fovea in fovea-splitting RRD suggests that surgical approaches may differ from those for other RRD types, potentially affecting visual outcomes.

Current surgical interventions for RRD primarily consist of scleral buckling (SB) and pars plana vitrectomy (PPV). A retrospective study by Wong et al. (11) demonstrated that in cases of macula-off primary RRD, the SB group had a significantly higher rate of functional success compared to the PPV ± SB group. Kawano et al.’s comparative analysis of PPV and SB for simple phakic macula-on retinal detachment showed no difference in best-corrected visual acuity (BCVA), but lower surgical failure and postoperative complication rates in the SB group (12). While both procedures have been extensively studied in patients with macula-on and macula-off RRD, the specific outcomes of SB and PPV in treating simple phakic fovea-splitting RRD remain understudied.

To determine whether the surgical technique affects anatomical and functional outcomes in fovea-splitting RRD, we conducted a comparative study examining the effects of PPV versus SB in managing primary simple phakic fovea-splitting RRD.

## Methods

We conducted a retrospective study focusing on patients with primary fovea-splitting RRD who were evaluated at the Eye Hospital of Wenzhou Medical University (WMU) between 2017 and 2023. The study was approved by the Institutional Review Board (IRB) of the Eye Hospital of WMU, which granted a waiver of informed consent due to the retrospective nature of the study. Adhering to the Declaration of Helsinki, we ensured strict protection of participant confidentiality and data security.

### Patients and data collection

This study included patients aged 18 to 55 years diagnosed with primary phakic fovea-splitting RRD who underwent SB or PPV procedures, with a minimum follow-up period of 6 months. Exclusion criteria were significant corneal opacification, advanced cataracts or vitreous hemorrhage, giant retinal tears, prior vitreoretinal surgery, PVR grades C or D, age-related macular degeneration, diabetic retinopathy, hereditary retinal conditions or vitreoretinal dystrophies, and extensive data loss.

Data were retrieved from the scientific research data platform of the Eye Hospital of WMU, including information on gender, age, laterality, duration of visual symptoms, extent and location of RD, size and number of retinal breaks, foveal status, logarithm of the minimum angle of resolution (LogMAR) BCVA, intraocular pressure (IOP), axial length (AL), lens status, type of surgery (PPV or SB), and the duration from OCT diagnosis to surgical intervention.

In the PPV group, a standard 23-gauge or 25-gauge vitrectomy was meticulously performed, with a focus on releasing traction at retinal breaks. Drainage of subretinal fluid was facilitated through preexisting breaks, and perfluoro-N-octane was used adjunctively when necessary. Retinopexy was achieved using endolaser around all retinal breaks, with cryotherapy employed when indicated. The choice of tamponade material, either gas or silicone oil, was at the discretion of the surgeon based on clinical judgment. In the SB group, a segmental scleral buckle procedure was conducted using segmental silicone sponges, and encirclage with encircling 240 bands and segmental silicone tires was applied when possible. Cryotherapy was used for retinopexy around all retinal breaks, and the drainage of subretinal fluid along with tamponade using air or expansile gas was performed according to the surgeon’s clinical decision.

**Figure 1:**
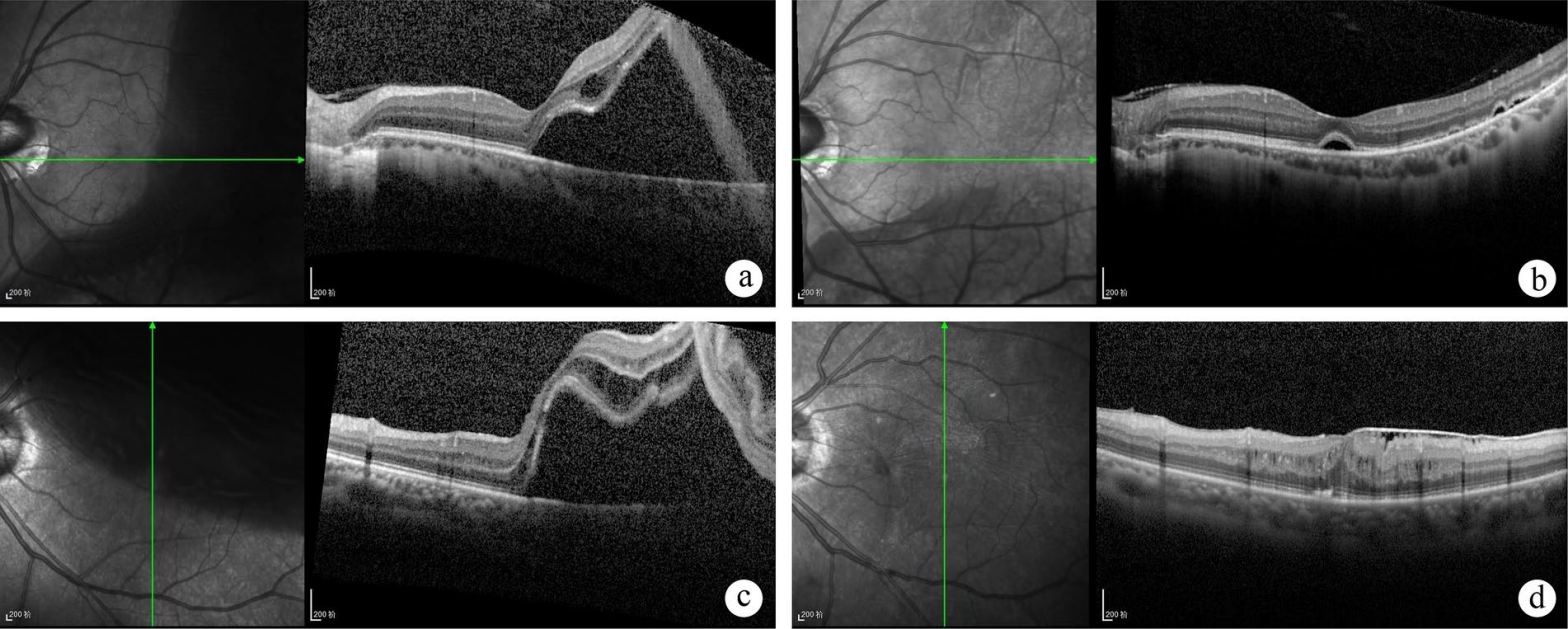
Optical coherence tomography (OCT) scans of patients diagnosed with foveal-split rhegmatogenous retinal detachment (RRD) on fundus examination at baseline and follow-up. A 36-year-old male patient is shown prior to scleral buckling (SB) surgery **(A)**. Postoperatively at 1 month, persistent subretinal fluid was observed **(B)**, which had resolved by the one-year follow-up. Visual acuity improved from 20/33 preoperatively to 20/25 postoperatively. A 55-year-old female patient presented with superior retinal detachment before undergoing pars plana vitrectomy (PPV) **(C)**. At the six-month postoperative follow-up, an epiretinal membrane, intraretinal fluid, disruption of the ellipsoid zone, and external limiting membrane (ELM) were observed **(D)**. Visual acuity remained at 20/67 preoperatively.

In accordance with the description by Lee et al. (10), fovea-splitting RRD was identified by preoperative OCT demonstrating retinal detachment affecting the foveal center and limited to within 750 μm of the foveal center (Figure 1). BCVA was recorded using logMAR units, with count fingers (CF), hand movements (HM), and perception of light (PL) substituted with the corresponding values of 2.10, 2.40, and 2.70, respectively. Functional success was defined as achieving a postoperative LogMAR BCVA of 0.3 or better at 6 months post-surgery; otherwise, it was considered functional failure. Primary anatomical success was determined by the achievement of retinal reattachment 6 months after the initial retinal detachment surgery. For eyes treated with silicone oil injection during the initial procedure, success was reassessed at 6 months post-silicone oil removal. OCT was utilized to confirm the presence of an epiretinal membrane (ERM) and cystoid macular edema (CME) during at least one follow-up visit, and persistent subretinal fluid (PSF) 1 month after the surgical intervention. ERM characterized by the presence of a single, hyper-reflective lines above the internal limiting membrane within the macular region. CME was defined as the presence of cystoid spaces within the inner or outer retina. PSF was identified as a clear space between the sensory retina and the retinal pigment epithelium (RPE) 1 month after the surgical intervention for retinal detachment. Two investigators (Zhu and Pan) evaluated the OCT images independently.

### Statistical analysis

Analyses were performed using the commercially available software package SPSS (version 22.0; SPSS, Inc., Chicago, IL, USA). Quantitative data are presented as means ± standard deviations (SD), while qualitative variables are expressed as percentages. The Kolmogorov–Smirnov test was used to determine whether continuous variables followed a normal distribution. For univariate analysis of categorical variables, the chi-squared test or Fisher’s exact test was applied, depending on the characteristics of the data. Continuous variables were analyzed using either the Wilcoxon rank-sum test or Student’s t-test, based on their distribution.

Predictors with a *p*-value of less than 0.2 from the univariate analysis were included in a multivariate logistic regression analysis (employing the forward stepwise method) to examine postoperative outcomes following retinal detachment surgery. The relationships between clinical factors and postoperative complications were quantified by calculating odds ratios (ORs) and their corresponding 95% confidence intervals (CIs) within the multivariate model. All statistical tests were two-sided, and a *p*-value of less than 0.05 was considered statistically significant.

## Results

### Preoperative clinical characteristics

A total of 101 eyes from 101 patients (57 males and 44 females), with a mean age of 37.4 ± 11.9 years (range, 18–55 years), were enrolled in the study. Of these patients, 51 were treated with SB, while the remaining 50 underwent PPV. Overall, patients treated with PPV were significantly older (45.5 ± 8.7 vs. 29.5 ± 9.0 years, *p* < 0.001) than those treated with SB and were more likely to have superior RRD (48.0% vs. 3.9%, *p* < 0.001). Additionally, they exhibited a shorter axial length (25.3 ± 2.0 vs. 25.8 ± 4.0 mm, *p* = 0.004), worse preoperative logMAR BCVA (1.09 ± 0.80 vs. 0.61 ± 0.51, *p* = 0.001), shorter duration of symptoms (21.7 ± 38.4 vs. 44.8 ± 81.3, *p* = 0.036), and lower preoperative IOP (11.6 ± 3.6 vs. 14.1 ± 2.9 mmHg, *p* < 0.001). Gender, extent of RD, number of tears, and time to surgery did not differ significantly between the two treatment groups. Baseline descriptive statistics and inter-group differences are presented in Table 1.

**Table 1:** Clinical characteristics of patients with fovea-splitting rhegmatogenous retinal detachment.

Baseline characteristics	Total (*N* = 101)	Scleral buckling (*N* = 51)	Pars plana vitrectomy (*N* = 50)	*p*
Age, years (SD)	37.4 (11.9)	29.5 (9.0)	45.5 (8.7)	<0.001
Male sex, *n* (%)	57 (56.4)	28 (54.9)	29 (58.0)	0.754
Preoperative BCVA, logMAR (SD)	0.85 (0.71)	0.61 (0.51)	1.09 (0.80)	0.001
Preoperative IOP, mmHg (SD)	12.9 (3.5)	14.1 (2.9)	11.6 (3.6)	<0.001
AL, mm (SD)	25.6 (3.1)	25.8 (4.0)	25.3 (2.0)	0.004
RD extent >2 quadrants, *n* (%)	8 (7.9)	2 (3.9)	6 (12.0)	0.160
Number of tears found preoperatively, *n* (%)				0.362
Single tear	45 (44.6)	25 (49.0)	20 (40.0)	
>1 tear	56 (55.4)	26 (51.0)	30 (60.0)	
RD configuration, *n* (%)				<0.001
Superior	26 (25.7)	2 (3.9)	24 (48.0)	
Equal	17 (16.8)	12 (23.5)	5 (10.0)	
Inferior	58 (57.4)	37 (72.5)	21 (42.0)	
Duration of symptoms, days (SD)	33.3 (64.5)	44.8 (81.3)	21.7 (38.4)	0.036
Time to surgery, days (SD)	5.0 (4.5)	5.3 (3.7)	4.8 (5.2)	0.082

SD = standard deviation; BCVA = best-corrected visual acuity; logMAR = logarithm of the minimum angle of resolution; IOP = intraocular pressure; AL = axial length; RD = retinal detachment.

### Clinical outcomes

Overall, 53 patients (52.5%) achieved a BCVA of 0.3 logMAR or better, indicating functional success. Furthermore, 65 eyes (64.4%) had a BCVA improvement of >0.2 logMAR, while 4 eyes (4.0%) experienced a BCVA loss of >0.2 logMAR. Table 2 compares clinical outcomes between the two patient cohorts. Patients who underwent PPV showed poorer postoperative BCVA (0.38 ± 0.35 vs. 0.25 ± 0.30, *p* = 0.029) and greater visual improvement (0.71 ± 0.80 vs. 0.36 ± 0.38, *p* = 0.024) compared to the SB group on univariate analysis. Follow-up data revealed that the PPV group had significantly higher rates of pseudophakia (82.0% vs. 3.9%, *p* < 0.001), and ERM (19.6% vs. 5.9%, *p* = 0.038). Conversely, PSF was more common in the SB group (74.5% vs. 22.0%, *p* < 0.001). There were no significant differences in follow-up duration, functional success, IOP exceeding 25 mmHg, postoperative CME, visual improvement greater than 0.2 logMAR, visual loss greater than 0.2 logMAR, or primary and final anatomical success.

**Table 2:** Clinical outcomes of patients with fovea-splitting rhegmatogenous retinal detachment.

Outcome	Total (*N* = 101)	Scleral buckling (*N* = 51)	Pars plana vitrectomy (*N* = 50)	*p*
Average length of follow up, days (SD)	731 (422)	674 (388)	789 (449)	0.252
Average Functional success, *n* (%)*	53 (52.5)	31 (60.8)	22 (44.0)	0.091
Average final postoperative BCVA, logMAR (SD)	0.31 (0.33)	0.25 (0.30)	0.38 (0.35)	0.029
Average visual improvement, logMAR (SD)	0.53 (0.65)	0.36 (0.38)	0.71 (0.80)	0.024
Visual improvement (>0.2 logMAR), *n* (%)	65 (64.4)	30 (58.8)	35 (70.0)	0.241
Primary anatomical success, *n* (%)^†^	95 (94.1)	48 (94.1)	47 (94.0)	1.000
Final anatomical success, *n* (%)^‡^	101 (100)	51 (100)	50 (100)	1.000
Silicone oil *in situ* at 6 months, *n* (%)	8 (7.9)	0 (0)	8 (16.0)	0.003
Pseudophakia during follow-up, *n* (%)	43 (42.6)	2 (3.9)	41 (82.0)	<0.001
Postoperative complications, *n* (%)				
IOP over 25 mmHg	47 (46.5)	19 (37.3)	28 (56.0)	0.059
Epiretinal proliferation	13 (12.9)	3 (5.9)	10 (20.0)	0.034
Cystoid macular edema	10 (9.9)	2 (3.9)	8 (16.0)	0.051
Persistent subretinal fluid	49 (48.5)	38 (74.5)	11 (22.0)	<0.001
Visual loss (>0.2 logMAR)	4 (4.0)	1 (2.0)	3 (6.0)	0.362

^*^Functional success was defined as postoperative logMAR BCVA of 0.3 logMAR or better at 6 months.

^†Primary anatomical success was defined as retinal reattachment at 6 months after 1 retinal reattachment procedure.^

SD = standard deviation; BCVA = best-corrected visual acuity; logMAR = logarithm of the minimum angle of resolution; IOP = intraocular pressure.

Multivariable logistic regression analysis was conducted to compare the two patient groups. The results are summarized in Table 3. Functional failure was more prevalent in patients with poorer preoperative visual acuity (OR = 1.099 per 0.1 logMAR worsening, 95% CI = 1.030–1.173, *p* = 0.004). Patients who underwent PPV were more likely to experience postoperative ERM formation (OR = 4.000, 95% CI = 1.030–15.534, *p* = 0.045) compared to those who underwent SB. Predictors of postoperative PSF included lower LogMAR visual acuity values (OR = 0.918 per 0.1 logMAR worsening, 95% CI = 0.846–0.996, *p* = 0.040), the presence of multiple tears (OR = 3.770, 95% CI = 1.292–10.997, *p* = 0.015), and undergoing SB (OR = 11.091, 95% CI = 3.839–32.041, *p* < 0.001). No significant differences were observed between the SB and PPV groups regarding postoperative CME (OR = 4.667, 95% CI = 0.939–23.192, *p* = 0.060 with SB as the reference group).

**Table 3:** Multiple logistic regression analyses assessing the correlation between clinical factors and postoperative outcomes after retinal detachment repair.

	Variable	OR	95% CI	*p*
Functional failure	Preoperative BCVA, per 0.1 logMAR higher	1.099	1.030–1.173	0.004
Occurrence of ERM	SB	Reference	Reference	0.045
	PPV	4.000	1.030–15.534	
Persistent subretinal fluid	Preoperative BCVA, per 0.1 logMAR higher	0.918	0.846–0.996	0.040
	Number of tears ≤1 tear	Reference	Reference	0.015
	Number of tears >1 tear	3.770	1.292–10.997	
	PPV	Reference	Reference	<0.001
	SB	11.091	3.839–32.041	

IOP = intraocular pressure; OR = odd ratio; CI = confidence intervals; BCVA = best-corrected visual acuity; logMAR = logarithm of the minimum angle of resolution; AL = axial length; SB = scleral buckle; PPV = pars plana vitrectomy.

### Subgroup analyses

To minimize the potential influence of inferior RRD and the timing of surgery on visual outcomes, we examined two subgroups of patients: (1) the inferior RRD subgroup, comprising individuals with the main causative tear between 3:00 and 9:00 clock hours in the detached retina, and (2) the promptly treated subgroup, consisting of patients who underwent surgery within 3 days. In the inferior RRD subgroup, individuals who underwent SB exhibited superior postoperative BCVA (0.24 ± 0.33 vs. 0.48 ± 0.33, *p* = 0.002) and a higher rate of functional success (68.6% vs. 26.3%, *p* = 0.003) compared to those in the PPV group. In a multivariable analysis, functional failure was associated with poorer preoperative visual acuity (OR = 1.159 per 0.1 logMAR worsening, 95% CI = 1.007–1.332, *p* = 0.039) and lower preoperative IOP (OR = 0.772 per 1 mmHg increase, 95% CI = 0.613–0.973, *p* = 0.028). But in the promptly treated subgroup, there were no significant differences in postoperative BCVA and rate of functional success, and no significant association was observed between functional failure and poorer preoperative visual acuity (OR = 1.084 per 0.1 logMAR worsening, 95% CI = 0.996–1.180, *p* = 0.061).

## Discussion

In this retrospective analysis of patients presenting with primary simple phakic fovea-splitting RRD, we compared the efficacy of SB and PPV. We found that both surgical approaches yielded comparable primary and final anatomical success rates, as well as similar functional success rates. However, the incidence of ERM formation was significantly higher in the PPV group.

Modern vitreoretinal surgical methods have notably improved anatomical success rates, and most studies that observed no significant discrepancies in single-operation success rates between PPV and SB in uncomplicated, primary RRD (11, 13, 14). However, the progress in achieving better visual results after surgery has been less pronounced. In Wong et al.’s study on macula-off primary RRD, SB showed a higher functional success rate (43.2%) compared to PPV ± SB (28.0%), with a statistically significant difference (*p* < 0.001) (11). Kawano et al. found a higher failure rate with PPV (15.3%) compared to SB (5.1%) in the treatment of simple phakic macula-on retinal detachment, yet observed no significant difference in BCVA changes between the procedures (*p* = 0.66) (12). Our study demonstrated a higher rate of functional success compared to Wong et al.’s research and similar anatomical success rates to Kawano’s study. This may be attributed to the intermediate position of fovea-splitting RRD, which lies between macula-off and macula-on RRD (4, 7, 10).

Several factors, including preoperative BCVA, duration of symptoms, foveal status, presence of PVR, extent of retinal detachment, timing of surgery, and vitrectomy-based procedures, have been linked to visual outcomes following surgical repair of detached retinas (2–6). Although the choice of surgical approach is largely determined by anatomical considerations and surgeon preference, the procedure itself may exert a considerable effect on visual outcome as a consequence of the anatomical modifications that occur during surgery (11).

For example, recent studies suggest that removal of the vitreous during PPV may also have a detrimental effect on visual acuity outcomes (11, 15). The presence of vitreous may inhibit the dissemination of inflammatory mediators toward the posterior pole by providing a neuroprotective environment for surrounding retinal cells and maintaining biochemical homeostasis (15, 16). Some current studies suggest that microstructural macular damage, such as reduced vessel density in the superficial and deep retinal plexuses and thinning of the ganglion cell layer-inner plexiform layer, is a direct consequence of PPV surgery, which may lead to incomplete visual recovery (17, 18). Furthermore, several potential intraoperative traumatizing factors could increase the loss of VA during PPV, including potential retinal ischemia, fluctuations in intraocular pressure, direct retinal manipulation, use of perfluorocarbon liquid, and light toxicity; all these factors may influence postoperative visual rehabilitation (15). Given the significant role of inflammation in the development of CME and ERM following PPV for RRD surgery, enhanced postoperative anti-inflammatory strategies, such as intravitreal injection of agents like Ozurdex, may effectively reduce the incidence of CME and ERM. Complete vitrectomy results in increased oxygen levels within the vitreous chamber, potentially leading to cataract progression. Therefore, limited vitreous removal that preserves the cortex, shielding the crystalline lens from excessive oxygen exposure, can reduce cataract formation (19).

In our univariate analysis, the SB group exhibited better final BCVA, which we speculate may be due to the younger age and better preoperative BCVA of patients in the SB group compared to those in the PPV group. As a result, in our multivariate analyses of all eyes, the sole factor associated with functional failure was preoperative BCVA, which is in line with previous research (2, 6). Additionally, our study found that patients in the PPV group experienced a greater visual improvement, which may reflect the more severe initial preoperative visual acuity in this group.

The higher rate of PSF observed in the SB group is a well-documented complication of this procedure, with incidence rates ranging from 15 to 83.1% as reported in the recent literature (20, 21). Scleral buckling surgery, for instance, has been associated with higher rates of PSF compared to vitrectomy, possibly due to the reliance on the RPE pump for fluid resorption and the potential for incomplete drainage during surgery (22, 23). However, the association between PSF and postoperative visual recovery is controversial. Most reports demonstrate that PSF may delay the recovery of the external limiting membrane and ellipsoid zone, which may contribute to slow visual recovery in the short-term but does not influence the final visual outcome (20, 24). There is concern that some cases with PSF may sustain photoreceptor damage, retinal displacement, or retinal fold formation, which are dangerous for VA recovery (25).

Furthermore, in our case series, 57.4% of the cases presented with inferior retinal detachment, and our subgroup analyses revealed that inferior RRD was associated with better visual outcomes in the SB group, suggesting that these factors may influence the choice of surgical approach. We believe that the inferior retinal detachment necessitates the use of silicone oil or perfluorocarbon liquid as tamponade agents during pars plana vitrectomy (PPV) surgery, which, however, can lead to significant complications and subsequently impact postoperative visual recovery (26). Our study emphasizes the importance of considering the specific location of retinal detachment when selecting the most appropriate surgical intervention. The findings suggest that SB may be a more advantageous option for fovea-splitting RRD, particularly in cases of inferior RRD, due to the anatomical location of the retinal break and the targeted support provided by SB.

This study does have several limitations. Firstly, the retrospective design of the study introduces potential biases that may impact the comparability of the SB and PPV groups. Secondly, although the sample size of our study is adequate for the analyses conducted, it is relatively modest, which may limit the generalizability of our findings and our ability to detect smaller differences between groups. Additionally, it restricts our capacity to perform a more detailed analysis of different RRD types, considering factors such as the location of retinal breaks, extent of detachment, superior and equal retinal detachment, and patient age. Thirdly, the OCT assessment is not conducted in a blinded manner, which could potentially introduce bias into the results. Lastly, our study population was confined to patients with primary phakic fovea-splitting RRD, and as such, our conclusions may not be extrapolated to pseudophakic or aphakic patients, or those presenting with more complex RRD cases, including those involving proliferative vitreoretinopathy or a history of vitreoretinal surgery.

In summary, we compared SB and PPV in patients with primary simple phakic fovea-splitting RRD. When compared with PPV, the primary anatomical success and final BCVA were similar; however, the incidence of ERM formation was significantly lower in the SB group. Therefore, careful consideration is needed when selecting the appropriate surgical technique for treating patients with phakic fovea-splitting RRD.

## Data Availability

The raw data supporting the conclusions of this article will be made available by the authors, without undue reservation.
